# Perceived competence and related factors affecting the development of the clinical competence of nursing students at two university sites in Namibia: a cross-sectional study

**DOI:** 10.1186/s12909-024-05729-z

**Published:** 2024-07-09

**Authors:** Takaedza Munangatire, Victoria Jacob, Nestor Tomas

**Affiliations:** University of Namibia, Maria Mwengere Street, P.O. Box 88, Rundu, Namibia

**Keywords:** Competence, Nursing student, Factors, Perceived, Development

## Abstract

**Background:**

Ensuring that nursing students graduate with the required clinical competence in nursing is a global challenge. To address this challenge, several studies have looked at various aspects of competency and competency development, however there is scanty evidence on factors affecting development of clinical competency in nursing students. Therefore the, purpose of this study was to investigate nurses’ perceived competence and related factors affecting the development of clinical competence of nursing students at two university sites in Namibia.

**Methods:**

A cross-sectional design was utilised. Simple random sampling was applied and 272 nursing students at two university campuses in Namibia were selected. An online questionnaire was used. Data were collected between April and May, 2022, over a period of six weeks, and were analysed using Statistical Package of Social Sciences (SPSS) version 27. Chi-square and Spearman correlations were used to assess the associations and correlations, respectively, among the variables. Logistic regression was used to assess the factors associated with the development of clinical competence using a *p*-value < 0.05 confidence interval.

**Results:**

Forty-seven percent (47%) of the students were found to be competent while more than half (53%) were not. A Chi-square test found a statistically significant difference between students studying at different campuses and between different year levels (*p* =  < .05). A regression analysis showed that nursing educators’ competence (β = .128; *p* = .36) had a positive influence on nursing students’ competence levels, unlike the mode of learning (β = -.140; *p* = .013), which negatively predicted the development of clinical competence. No significant relationship was found between the development of clinical competence and teaching approaches, assessment, feedback, constructive alignment, theory–practice gap and reflective practice (*p* = ˃.05).

**Conclusion:**

Educator’s competence levels and the mode of learning were the two major factors that were more likely to influence the development of clinical competence among nursing students. Therefore, it is recommended that nursing training institutions prioritise the development of educators’ competence and apply various modes of learning to enhance development of nursing students’ competence.

**Supplementary Information:**

The online version contains supplementary material available at 10.1186/s12909-024-05729-z.

## Background

Competence is the ability to perform required skills at an expected performance level or standard, resulting in desirable outcomes [1, 2]. While this description may seem simple, the enactment of ‘competence’ is anything but. Indeed, there are many facets to it. First, competence integrates the knowledge, skills, and attitudes that should be displayed during clinical performance in various healthcare situations [3]. Second, competence has an element of continuity, meaning that it is a continuous process of acquiring necessary sets of skills and updating one’s knowledge, skills, and attitudes in response to the changing clinical environment [4]. Ultimately, one cannot claim to be competent in every situation and context. Clinical contexts and circumstances often present new challenges requiring one to approach their practice in new ways [2, 5]. In the context of nursing education, competence is complex, and its development is expected to start at the beginning of nurses training [6]. Researchers argue that clinical competence should be the ultimate endpoint of nursing education, with nurses entering the field of healthcare with the ability to practice independently, safely, and effectively [7, 8].

The ultimate purpose of nursing education is to facilitate students’ learning, which leads to the development and graduation of competent nurses [9–11]. However, competence can only develop if a myriad of factors work in synergy to support learning that is directed toward the development of competence. To date, there is no conclusive evidence of how to optimally facilitate the development of nursing students’ clinical competence. There is also a lack of clarity about which factors are significant in supporting competence development. Bvumbe and Mtshali [12] argued that nursing education has an important role to play in producing fully competent nurses. To produce these competent nurses, nursing education programmes should be fit for purpose [13], and one way to achieve this is to determine which critical factors support the development of competence. If the factors associated with the development of competence are established, then there is potential that effective nursing education can be implemented, resulting in clinically competent nurses [13].

This study aims to respond to several calls demanding better healthcare in Namibian hospitals. With the growing demand for accountability and the improvement of patient care [14], nursing education research needs to explore the concept of competency development to produce competent nurse graduates. Currently, there is evidence showing that some practising nurses and newly graduated nurses are not clinically competent [15]. Because nurses are the backbone of the healthcare system, the search for evidence to support their development of competence is needed to avoid poor nursing care and healthcare outcomes [16]. Nursing students need a learning environment that promotes the development of clinical expertise and the application of theoretical knowledge [12, 17]. However, to create such a supportive learning environment, there is a need to understand nursing students’ perceptions of the different factors that influence their development of clinical competence. Also, given the complexity of illness globally, the demand for healthcare professionals, and the globalisation of healthcare services, the expectation is that nursing graduates are clinically competent for practice upon completion of their nursing education programme [18–21]. However, there are questions about the competence of nursing graduates globally and Namibia is no exception [12, 22]. Hence, there is a need to explore how different factors impact the development of nursing students’ clinical competency.

Additionally, the dynamic nature of competence demands that there is a better understanding of the various aspects at play in the development of clinical competence. For example, how a nurse’s knowledge, understanding, judgment, cognitive, technical, psychomotor, and interpersonal skills are developed and interlinked to produce competency [23]. Also, the nature of the healthcare environment is such that it is ever-changing. There are regularly, for example, new treatments mediated by technology, emerging professional responsibilities, and healthcare challenges, which mean that competence is not permanent [16]. For this reason, it is important to monitor changes in nursing students on an ongoing basis [24], since without the necessary levels of competence, much can go wrong. Nurses who lack clinical competence are prone to causing care errors, which may adversely impact patient safety [25]. In addition, poor levels of competence can lead to dissatisfaction and feelings of frustration for both nurses and patients [26, 27].

The literature demonstrates that the measure of the clinical competence of nurses or nursing students relies on self-reported scales such as the Professional Nurse Self-Assessment Scale (ProffNurse SAS) [28], the Nurses' Professional Competence Scale Short Form (NPCS-SF) [29], the Nurse Competence Scale (NCS) [30], and the Nursing Student Competence Scale [31]. Taylor [32] also advocate for the use of self-assessments considering them suitable for the evaluation of the clinical competence of nursing students. To more accurately complete these self-assessments, however, Munangatire and McInerney [3] demonstrated that students should understand the concept of competence, making it possible for them to accurately judge their levels of clinical competence. There are some suggestions that students or nurses overestimate their clinical competence [33],but one study shown that at least 60% of students who completed such self-assessment questionnaires considered themselves incompetent [2]. This result is mirrored in a study by Egilsdottir [8] who found that students generally described their competence as needing improvement. Therefore, until objective measures are established to authentically measure nurses’ clinical competence, self-reported measures remain relevant, despite the risk of over or underestimation.

The development of competence has been investigated in many studies from different angles.

For example, in a study by Egilsdottir [8], socio-demographic factors such as age, year of study, and gender did not affect students’ levels of clinical competence. Rather, factors such as self-efficacy and professional interest were reported as vital in supporting the development of clinical competence and professionalism [34, 35]. Gemuhay [10] had similar findings with student-related factors such as self-efficacy, amongst others, being significantly associated with the nursing students’ clinical performance.

Clinical placement is also an important factor in nursing students’ learning and development of clinical competence. Research on nursing students’ clinical performance shows that placement-related factors were reported as influential in the performance of nursing students [10]. The role of the clinical learning environment in affecting nursing students’ development of clinical competence was further demonstrated in a recent study by Yu [17]. However, despite the results of the studies above, it is not clear which aspects of the clinical environment improve or hinder the development of clinical competence and to what extent either may be the case. Current literature indicates that despite the nature of the clinical placement, some students do develop the required competencies while others fail to do so [15].

In addition to the clinical environment, factors related to nursing educators have also been consistently linked to nursing students’ clinical competence. According to Seo and Park [34], teaching effectiveness significantly affected nursing students’ levels of clinical competence. Similarly, a study published a year later suggested that the level of nursing students’ clinical practice competence was greatly determined by the quality of the clinical teachers [35]. Research also shows that students acquire clinical competencies most effectively when clinical teachers are supportive and motivate students to learn [36]. Characteristics such as these enable a good supervisory relationship to develop and create a positive learning atmosphere, which may lead to the improved development of clinical competencies [37]. Consequently, the role of nursing educators in the development of the clinical competence of nursing students cannot be underestimated.

Further cementing the role of nursing educators in the development of nursing students’ clinical competence is evidence suggesting that even student-related factors need the input from an educator to influence the development of competence. Uemura [38] highlighted that nursing students developed good levels of competence when they learned through participation, reflection, practice, and feedback. Such activities can only be effective if supported by the educator. Comparably, constructive feedback and assessment orientation, which are factors inherently dependent on the educator, were found to be significantly associated with perceived clinical competence [2]. Constructive feedback, for example, can help students improve their metacognition skills, which contribute to the development of their problem-solving competence [39].

Inherent to the nursing education environment in Namibia, is the shortage of nurse educators, nursing staff, training resources, and an increasing nursing student population. Nursing students’ lack of clinical competence remains a challenge for hospital managers, nursing regulating bodies, and the government [40]. This lack of clinical competence remains the key factor responsible for poor quality nursing care and low client satisfaction [41, 42]. Yet despite these concerns, it remains unclear which factors affect nursing student’s development of clinical competence in Namibia. There is limited evidence on nursing students’ perceived level of competence and the factors influencing development of competence. Subsequently, there is a need to investigate students’ perceptions of their development of clinical competence and of which factors influence this development. The purpose of this study was to investigate nurses’ perceived competence and related factors affecting the development of clinical competence of nursing students at two university sites in Namibia.

## Methods

### Design

This is a quantitative study that used a descriptive, cross-sectional study design. In this cross-sectional study, the students’ perceived level of competence was the independent variable and the factors related to the development of clinical competence were the dependent variables.

### Study setting

The university at which the study took place has four campuses offering nursing degrees across Namibia. This study, however, was only completed on two of the four campuses which the researchers had reasonable access to and had the largest population of students. The students are expected to attend two weeks of theoretical classes facilitated by lecturers and two weeks of clinical learning at hospitals and clinics. These training hospitals and clinics also accommodate students from other universities and colleges of nursing. Nurses are the primary facilitators of learning as they are with the students all the time. It is during this time that nursing students are expected to develop clinical competency. While nurses are involved in facilitating learning, their role in assessing students in high-stakes examinations is limited to non-existent.

### Participants

All nursing students at the university’s two campuses (A; *N* = 451 and B; *N* = 399) (*N* = 850) were invited to take part in the study. In terms of sampling, the population was clustered into campus A and B, then at both campuses total census sampling was applied to include all nursing students enrolled in the degree program. Subsequently proportional stratified random sampling was used because it enabled the representation of each segment of the population. In stratified random sampling, the student population (*N* = 850) was divided into subgroups so that each element of the population belonged to one stratum only. Then, within each stratum, convenience sampling was applied until the required sample size was reached. The student population included 1st (N = 212) 2nd (N = 196), 3rd (*N* = 212) and 4th (*N* = 230) year nursing students. The researcher randomly approached the students in all cohorts until the targeted sample of 272 (campus A; *N* = 144 and campus B; *N* = 128) was reached. Slovin’s formula was used to determine the sample size which was calculated proportionally per campus 272 and per year of study (1st;*n* = 66, 2nd;*n* = 63, 3rd;*n* = 66,4th *n* = 72). The inclusion criteria were all nursing students enrolled in the nursing program at the two campuses and exclusion criteria were all nursing students enrolled in nursing programs other than the degree program only.

### Measures

The questionnaire used in this study was developed by the researchers and was based on clinical competence development literature. During the questionnaire development, educators were consulted on factors related to development of competence. After compiling the initial draft of the questionnaire, it was shared with three experts (nurse educators and researchers) for their input to ensure face and content validity. Further validation was enhanced through a pilot study. The questionnaire’s Cronbach Alpha coefficient was 0.75 based on the pilot test data and increased to 0.889 after actual data collection. Pilot study was conducted among 20 participants (10 from each campus) and these were excluded from the main study. Following data reduction through exploratory factor analysis, the score was 0.924, suggesting a good level of reliability. This score also means that the items were related and most likely measured the same construct.

The final questionnaire consisted of 52 items related to the participants’ demographic data and possible factors affecting their development of clinical competence. Section A comprised five questions focusing on the demographic data of the participants (age, gender, place of study, year of study, and clinical competency status). Section B consisted of 47 questions focusing on the following issues: learning approach, teaching approach, understanding of competence, assessment, feedback, learning outcomes, constructive alignment, theory–practice gap, reflection, mode of learning, resources, and teachers’ competence. Participants were asked to rate the extent to which each of the stated factors affected the development of competence using a Likert scale ranging from 1 = Never to 5 = Always. It was estimated that the questionnaire will be completed in 20–30 min.

### Data collection procedure

The researcher obtained permission from the University of Namibia’s research committee to conduct the study. Thereafter, the researcher approached nursing students face-to-face and explained the purpose of the study. The researcher also requested to be added to class WhatsApp groups through class representatives for each year of study in each campus. Via the WhatsApp groups, an online questionnaire was shared and interested students indicated their consent, as required, on the first page of the questionnaire. After consenting to take part in the research, they continued to complete the questionnaire. An online questionnaire was chosen as it allows for easy access; some students were completing online learning at the time of data collection and thus could not come to the campuses. Students took approximately 20 min to complete the questionnaire using their smartphones (or the researcher’s phone if necessary and if on campus). The students were able to complete the questionnaires at a time that was convenient to them. They were reminded to participate over weekends as this was perceived to be a less busy time. The aim of the questionnaire was to collect information on factors affecting the development of the clinical competence of nursing students, as well as their demographic data. Data was collected over a period of six weeks from the 17th of April to the 30th of May, 2022.

### Data analysis

Data were analysed using SPSS for Windows version 27.0 applying both descriptive and inferential statistics. Categorical variables were presented as frequencies and percentages (gender, campus, year of study) while continuous variables were analysed using mean and standard deviation (age, competency level). While age was measured as a continuous variable on the questionnaire, it was categorised during data analysis into three categories. Inferential statistics, specifically Chi-square cross-tabulations was performed to determine if there was a significant association between competent and non-competent students and their age groups, gender, campus, and year of study. The level of significance was 0.05 (two-tailed).

To reduce the multidimensionality of the data, exploratory factor analysis was applied [43]. The Kaiser Meyer-Olkin (KMO) test was used as a criterion for assessing the adequacy of the sample and the suitability of the data for exploratory factor analysis (EFA). The KMO value was 0.883, which exceeded the acceptable threshold of 0.5. Bartlett’s test of sphericity was also statistically significant (*p* < 0.001; Chi-square = 4464.80, df 946), thereby justifying the application of EFA. Factor analysis was conducted on all variables in section B of the questionnaire to explore the interrelationships between variables, and to identify the variables that were determined as having an influence on the development of clinical competence. To determine the number of factors to be included in the factor analysis, the eigenvalues were examined. Of the 47 initial items/factors in section B of the questionnaire that were purported to influence the development of nursing students’ competence, 11 factors were extracted as they had eigenvalues greater than one from the EFA as shown in Table 1. The 11 factors that were extracted explain 59.31% of the total variance.

**Table 1:** Total variance explained

Factor	Initial eigenvalues	% of Variance	Cumulative %
1	11.315	25.716	25.716
2	2.727	6.197	31.913
3	1.958	4.450	36.363
4	1.604	3.645	40.007
5	1.430	3.250	43.257
6	1.329	3.022	46.278
7	1.294	2.940	49.219
8	1.233	2.801	52.020
9	1.098	2.496	54.516
10	1.089	2.476	56.992
11	1.020	2.318	59.310

The factors were named to identify the broad construct contributing to the inter-correlation. Based on the meaning of the inter-related variables, the factors were named as shown in Table 2 below.

**Table 2:** Name of extracted factors

Factor	Name
1	Subject matter expertise
2	Alignment and authenticity of content
3	Authenticity of assessments
4	Assisted active reflective practice of skills
5	Deep understanding of competence
6	Personal active reflective practice
7	Blended learning
8	Strategic teaching and learning
9	Theoretical learning/teaching
10	Passive reflection of learning
11	Availability of clinical teachers

### Ethics approval and consent to participate

The university School of Nursing Ethics Committee approved this study (SoN 21/2022). Participants were informed of their choice to voluntarily participate. Participants were also guaranteed confidentiality, anonymity, beneficence, justice, and the right to withdraw from the study (i.e., their right to stop answering the questionnaire even if they had already started it). The participants were informed that the link was not associated with any identifying details and did not ask them for any identifying information. All the responses were received on the researchers’ database and were only accessible through passwords. Informed consent was obtained by providing the participants with information about the study. This formed the first part of the online questionnaire where participants were asked to tick the “Agree” box, which acted as signed consent to participate. Without ticking the box to consent to participate, students could not proceed to the actual questionnaire.

## Results

### Demographic data

The target sample was 272. Having received 272 responses, the study thus had a 100% response rate. Table 3 below shows that of the 272 respondents, the majority were females, 76% (*n* = 206), and 24% (*n* = 66) were males. The mean age of the respondents was 22.62 (± 3.87), of which 81% (*n* = 220) were aged between 20 and 29 years old, 11% (*n* = 31) were less than 20 years old and 8% (*n* = 21) were above 30 years old. With regard to the year of study, 27% (*n* = 72) were in their fourth year. Respondents in their third and first years each accounted for 25% (*n* = 66) of the total sample, and 23% (*n* = 63) of the respondents were in their second year. A total of 53% (*n* = 144) of the respondents were from campus A and 47% (*n* = 128) were from campus B. Table 3 below shows the frequency distribution across the demographic data. The self-reported perceived competence assessment showed that 52.9% of the students considered themselves competent while 47.1% indicated that they were not.

**Table 3:** Frequency distribution across demographic data (*n* = 272)

Variable	Frequency	Percentage%
**Age groups**		
Less than 20 years	31	11%
20–29 years	220	81%
30 and above	21	8%
**Gender**		
Male	206	76%
Female	66	24%
**Campus**		
A	144	53%
B	128	47%
**Year of study**		
First year	68	25%
Second year	63	23%
Third year	69	25%
Fourth year	72	27%
**Competency**		
Not competent	144	52.9
Competent	128	47.1
**Total**	**272**	**100%**

A Chi-square analysis was performed to determine if there was a significant association between competent and non-competent students and their age groups, gender, campus, and year of study. Table 4 above shows a strong association between the students’ self-reported perceived competence levels and their year of study (*p* = 0.021) as well as their place of study (*p* = 0.001). This suggests that students studying at campus A were more likely to report being competent than those on campus B. The cross-tabulations further showed that fourth-year students were more likely to report being competent than students in their first, second, or third years of study. However, no significant association was found between students’ self-reported perceived competence and their age groups (*p* = 0.364) or gender (*p* = 0.764).

**Table 4:** Chi-square Cross Tabulation: association between self-reported competence and other variables

Variable	Competent	Not competent	Total	*P* value
**Age group**				
Less than 20 years	14	17	31	.364
20 to 29 years	101	119	220	
30 years and above	13	8	21	
**Gender**				
Male	30	36	66	.764
Female	98	108	206	
**Campus**				
A	82	62	144	.001
B	46	82	128	
**Year of study**				
First year	22	46	68	.021
Second year	31	32	63	
Third year	33	36	69	
Fourth year	42	30	72	

As shown in Table 5 above, a logistic regression analysis was conducted to predict the factors that are more likely to influence the development of competence. The factors (independent variables) tested were: subject matter expertise, alignment and authenticity of content, the authenticity of assessments, assisted active reflective practice of skills, deep understanding of competence, personal active reflective practice, blended learning, strategic teaching and learning, theoretical learning/teaching, and passive reflection of learning. An educator’s level of competence (β = 0.128; *p* = 0.36) had a positive significant influence on the students’ development of clinical competence, whereas the mode of learning (β = -0.140; *p* = 0.22) negatively influenced students’ development of competence.

**Table 5:** Regression table: logistic regression analysis

**Model**	**Standardised coefficients**					**95.0% Confidence interval for B**	
	**B**	**Std. Error**	**Beta**	**t**	**Sig**	**Lower Bound**	**Upper bound**
(Constant)	1.471	.030		49.244	< .001	1.412	1.529
Subject matter expertise	.045	.036	.078	1.267	.206	-.025	.115
Alignment and authenticity of content	-.013	.036	-.023	-.373	.710	-.084	.057
Authenticity of assessments	.056	.036	.094	1.549	.122	-.015	.128
Assisted active reflective practice of skills	.018	.038	.029	.468	.640	-.056	.091
Deep understanding of competence	.029	.038	.047	.773	.440	-.045	.104
Personal active reflective practice	-.040	.037	-.065	-1.075	.283	-.113	.033
Mode of learning	-.082	.036	-.140	-2.308	.022	-.153	-.012
Educator’s competence	.084	.040	.128	2.111	.036	.006	.162
Theoretical learning/teaching	.032	.038	.052	.853	.395	-.042	.106
Passive reflection of learning	-.001	.041	-.002	-.026	.979	-.081	.079
Availability of clinical teachers	.030	.040	.046	1.267	.446	-.048	.109

## Discussion

The results from our research showed that there are 11 factors that can influence the development of clinical competence. Two factors, mode of learning and teacher competence, were more likely to predict the development of nursing students’ clinical competence.

### Perceived competence

The students on one campus reported higher competence levels compared to the students on the other campus. This result could be interpreted in many ways. It could be that students on one campus are more confident in themselves and feel they are thus competent. Alternatively, this result could also mean that students are either over- or under-rating themselves. Indeed, other similar studies have shown that nursing students tend to either over-rate or under-rate their performance levels [33, 44].

Mixed results have also been found regarding the impact of the place of study on students’ levels of clinical competence. Some studies reported that a student’s place of study has a significant impact on the development of competence [32, 45, 46]. Other studies, however, found that the place of study has no effect on the development of student competence [47, 48]. In this study, the students’ levels of clinical competence were found to be associated with the year of study. Fourth-year students reported high levels of competence compared to the rest of the sample. This result is supported by Kang [49], Amsalu [40], and Sahin [50] who all reported that nursing students’ competence improved as they progressed through their years of study. This makes sense since, logically, as students move from one year of study to the next, they accumulate more knowledge, skills, and experience that ultimately increase their competence. However, it is important to note that it is not just the passing of the years that results in increased levels of competence, but actual teaching and learning experiences.

### Perceived competence and associated factors

No significant association was found between the students’ age groups and their perceived levels of competence, results reflected with studies by Gemuhay [10]. In a study completed by Amsalu [40], age groups was also found to not have a significant influence on the levels of nursing students’ clinical competence. However, a study by Yamamoto [51] et al. did find that age affects the level of clinical competence of nursing students. This contrary result is also in line with a study by Amsalu [40] who found that age is significantly associated with the clinical competence of nursing students. The research is thus mixed when it comes to the effect of age on nursing students perceived clinical competence. Thus, the present study’s result may be explained by the lack of great variance in the age of undergraduate student participants.

### Factors related to the development of competence

Evidence from this study reveals two distinctive factors that influence nursing students’ perceived levels of clinical competence. These two factors are the educator’s competence and the mode of learning. In keeping with the literature, the competence levels of the nursing educators in this study influenced the students’ clinical competency (*p* =  < 0.05) [34, 48]. This means that the more competent the teacher is, the more competent the students become. The results from the current study also revealed that the mode of learning had a statistically negative effect on the development of students’ competence. Different learning methods, such as a face-to-face or online mode, affect the development of nursing students’ competency. These results concur with Abdulla [52] who found that students who learn through the face-to-face mode reported higher levels of clinical competency compared to students who were taught using an online mode.

Students’ reflection on their learning was found to have no influence on the development of competence. This finding is contrary to Pai [53] and Sodersved [54] who found a significant positive association between reflection and the development of students’ competence. The findings of this study are also contrary to a study by Wihlborg [55] who reported that when students do have the capacity and opportunity to reflect on their professional and personal practice, it can have a significant impact on competence development. This indicates that reflection is a crucial component of professional competence, learning, and growth.

The teaching approach did not influence the students’ development of competence in this study. However, other studies have shown that the development of competence is influenced by the way the students are taught. According to Saud and Chen [56], how students are taught greatly influences the development of their competence. In addition, when the instructor is experienced and skilled in the subject matter they are teaching, when they use the coaching technique to aid learning, and when they serve as an example for their students, then the students’ knowledge, critical thinking abilities, and confidence levels increased [57].The contrary results of this study could be explained by the possibility that the students are highly self-directed in their learning and rather choose to take responsibility for the development of their clinical competence, rather than let their teachers drive their learning.

While feedback was not associated with competence in this study, prior studies have found that positive feedback influences the development of the clinical competence of nursing students [58, 59]. The differences in the findings could be attributed to self-efficacy, differences in year levels, and the resilience of nursing students in different settings [60]. Also contrary to this study’s findings, other researchers argue that self-assessment plays an important role in assisting educators to ensure constructive teaching alignment between what content students need to know and how knowledge of this content can be gained [61]. Given that teaching and learning have shifted from the dominant face-to-face mode to the online and blended modes, the effectiveness of constructive alignment in the development of clinical competence needs further investigation, particularly in the Namibian context owing to a current lack of evidence.

Clearly, this study highlights that the development of nursing students’ clinical competence is associated with the campus and with year level. Interestingly, an educator’s competence and the mode of teaching also emerged as factors that can influence students’ development of clinical competence. Therefore, it is recommended that nursing training institutions prioritise the recruitment of competent educators.

### Limitations

The results of this study were based on self-reported data hence, the results do not necessarily confirm the students’ actual level of clinical competency. The participants’ responses about which factors can influence the development of their competency were also based on individual students’ experiences, which may vary from student to student, and context to context. However, examining these issues from the students’ perspectives is nevertheless important considering that student learning behaviour can be better influenced if their perceptions about the teaching and learning process are known.

## Conclusion

Educator’s competence levels and the mode of learning were the two major factors that were more likely to influence the development of clinical competence among nursing students. Based on this, it can be argued that educators play the most significant role in developing the competence of nursing students. Educators can help to shape and direct students’ learning so that they can better develop the necessary clinical competence. Therefore, it is recommended that nursing training institutions prioritise the development of educators’ competence and apply various modes of learning to enhance development of nursing students’ competence.

### Supplementary Information

Supplementary Material 1.

## Data Availability

The data used and analyses in this study are available upon reasonable request to the corresponding author.

## Abbreviations

KMO: Kaiser Meyer-Olkin
EFA: Exploratory factor analysis
SPSS: Statistical Package of Social Sciences
WHO: World Health Organisation
ProffNurse SAS: Professional Nurse Self-Assessment Scale
NPCS-SF: Nurses' Professional Competence Scale Short Form
NCS: Nurse Competence Scale
