# Supplementary material for: Perceived competence and related factors affecting the development of the clinical competence of nursing students at two university sites in Namibia: a cross-sectional study
Source: BMC Med Educ. 2024 Jul 9;24:743. doi: 10.1186/s12909-024-05729-z (PMC11234757; doi:10.1186/s12909-024-05729-z)
Supplement: Supplementary file 1 — Supplementary Material 1. [file 12909_2024_5729_MOESM1_ESM.pdf]

QUESTIONNAIRE

My name is Victoria Jacob and I am a postgraduate nursing student carrying out a research study entitled

"Factors affecting the development of clinical competence among nursing students at the University of Namibia."

Ethical clearance for this research study has been obtained from the University of Namibia School of Nursing Ethical Committee. Ethical clearance number: SoN 21/2022.

You are invited to participate in this study because you are a nursing student at the University of Namibia, Windhoek or Rundu campus.

This study is open to all nursing students from first year to fourth years at Windhoek and Rundu campus and you have the right to withdraw from participating in the study anytime you wish to do so.

Confidentiality is maintained as data collected is only shared between the researcher and the supervisor.

Anonymity: Nowhere in the questionnaire will you be requested to indicate your identity. Anonymity is assured as no names of the participants will be linked to the data collection. No identifying information will be associated with your responses.

Contact details
Takaedza Munangatire 0817824677 (Supervisor)
Victoria Jacob 0812213311 (Student)

* Indicates required question

Personal factors

Please indicate your response by choosing one of the given options and answer all the questions.

1. Age *

____

2. Year of study *

Mark only one oval.

- 1st
- 2nd
- 3rd
- 4th

3. Gender *

Mark only one oval.

- Male
- Female

4. Campus *

Mark only one oval.

- Windhoek
- Rundu

5. Choose one option that best describes your level of competence. I am competent if I am able to; *

Mark only one oval.

- Complete any given task
- Pass an assessment on any given nursing task
- Apply the theory into practice
- Practice according clinical guidelines and standards
- Nurse patient with best possible positive outcome

Other factors

1- Not at all
2- Rarely
3-Sometimes
4. Most of the time
5. Always

6. Studying for the purpose of memorisation improves students' competence *

Mark only one oval.

	1	2	3	4	5	
Not	◯	◯	◯	◯	◯	Always

7. Studying for the purpose of using the information in real life clinical practice improves students' competence. *

Mark only one oval.

	1	2	3	4	5	
Not	◯	◯	◯	◯	◯	Always

8. Lecturers who transmit information to students help improve students' competence. *

Mark only one oval.

	1	2	3	4	5	
Not	◯	◯	◯	◯	◯	Always

9. Lecturers who facilitate students acquisition deep understanding of information improve students' competence. *

Mark only one oval.

	1	2	3	4	5	
Not	◯	◯	◯	◯	◯	Always

10. Lecturers who facilitate student application of information into real clinical practice improve students 'competence. *

Mark only one oval.

	1	2	3	4	5	
Not	◯	◯	◯	◯	◯	Always

11. Students who understand competence beyond passing assessments develop competence better than those who understand competence as passing assessments *

Mark only one oval.

	1	2	3	4	5	
Not	◯	◯	◯	◯	◯	Always

12. Students who understand competence as positive patient outcome develop competence better than those who understand competence as not related to patient outcomes *

Mark only one oval.

	1	2	3	4	5	
Not	◯	◯	◯	◯	◯	Always

13. Students who understand competence as practice meeting clinical guidelines and standards develop competence better than those who understand competence as not related to clinical standards *

Mark only one oval.

	1	2	3	4	5	
Not	◯	◯	◯	◯	◯	Always

14. Students who understand competence beyond just performing and completing task develop better competence than those who understand competence as completing a task. *

Mark only one oval.

	1	2	3	4	5	
Not	◯	◯	◯	◯	◯	Always

15. Students who understand competence beyond application of theory to practice develop competence better than those who understand competence as application of theory to practice. *

Mark only one oval.

	1	2	3	4	5	
Not	◯	◯	◯	◯	◯	Always

16. Assessments that requires students to recall the information and skills they memorised helps the development of competence. *

Mark only one oval.

	1	2	3	4	5	
Not	◯	◯	◯	◯	◯	Always

17. Assessment that require students to apply theory to real life clinical situations helps development of competence. *

Mark only one oval.

	1	2	3	4	5	
Not	◯	◯	◯	◯	◯	Always

18. Assessment that require students to critically think and make decisions based on real life clinical situation helps development of competence *

Mark only one oval.

	1	2	3	4	5	
Not	◯	◯	◯	◯	◯	Always

19. Assessments in simulation helps the development of competence *

Mark only one oval.

	1	2	3	4	5	
Not	◯	◯	◯	◯	◯	Always

20. Assessment in real life clinical situations helps the development of competence *

Mark only one oval.

	1	2	3	4	5	
Not	◯	◯	◯	◯	◯	Always

21. The development of competence depends on the number of hours spent in clinical practice. *

Mark only one oval.

	1	2	3	4	5	
Not	◯	◯	◯	◯	◯	Always

22. The development of competence depends on the number of times the student has performed a skill *

Mark only one oval.

	1	2	3	4	5	
Not	◯	◯	◯	◯	◯	Always

23. The development of competence depends on the student's thinking about the procedure when performing the procedure *

Mark only one oval.

	1	2	3	4	5	
Not	◯	◯	◯	◯	◯	Always

24. The development of competence depends on the students' reflective thinking about the procedure after performing the procedure *

Mark only one oval.

	1	2	3	4	5	
Not	◯	◯	◯	◯	◯	Always

25. The development of competence depends on the feedback that shows what the students has done wrong or right proved by signing or not signing the log book *

Mark only one oval.

	1	2	3	4	5	
Not	◯	◯	◯	◯	◯	Always

26. The development of competence depends on the feedback that shows what and why the students' performance is wrong or right provided in addition to signing logbook *

Mark only one oval.

	1	2	3	4	5	
Not	◯	◯	◯	◯	◯	Always

27. The development of competence depends on the feedback that shows what student can do differently to improve future performance provided in addition to signing logbook *

Mark only one oval.

	1	2	3	4	5	
Not	◯	◯	◯	◯	◯	Always

Keep going

28. The provision of learning outcomes that based on what the student should know helps in the development of competence. *

Mark only one oval.

	1	2	3	4	5	
Not	◯	◯	◯	◯	◯	Always

29. The provision of learning outcomes that are based on what the student is expected to do in real practice helps the development of competence. *

Mark only one oval.

	1	2	3	4	5	
Not	◯	◯	◯	◯	◯	Always

30. Facilitation learning content aligned with the learning outcomes improves the development of competence *

Mark only one oval.

	1	2	3	4	5	
Not	◯	◯	◯	◯	◯	Always

31. Assessments activities that are aligned to the learning outcomes and content improves the development of competence. *

Mark only one oval.

	1	2	3	4	5	
Not	◯	◯	◯	◯	◯	Always

32. Teaching content that reflects what happens in real life clinical situation improves development of competence. *

Mark only one oval.

	1	2	3	4	5	
Not	◯	◯	◯	◯	◯	Always

33. Teaching content that reflects what is in the prescribed textbooks improves development of clinical competence. *

Mark only one oval.

	1	2	3	4	5	
Not	◯	◯	◯	◯	◯	Always

34. Encountering differences between the theoretical content and real-life practice improves development of competence *

Mark only one oval.

	1	2	3	4	5	
Not	◯	◯	◯	◯	◯	Always

35. Encountering similarities between what I learnt in class with what is in the real-life practices improves the development of competence. *

Mark only one oval.

	1	2	3	4	5	
Not	◯	◯	◯	◯	◯	Always

36. Describe the learning process in detail explaining what I did during learning helps development of competence *

Mark only one oval.

	1	2	3	4	5	
Not	◯	◯	◯	◯	◯	Always

37. Reflect upon what I thought and felt during the process of learning something helps development of competence *

Mark only one oval.

	1	2	3	4	5	
Not	◯	◯	◯	◯	◯	Always

38. Evaluation of what I did right and what helped me do it right helps development of my competence *

Mark only one oval.

	1	2	3	4	5	
Not	◯	◯	◯	◯	◯	Always

39. Evaluation of what I did not do right and what caused it to go wrong helps development of my competence *

Mark only one oval.

	1	2	3	4	5	
Not	◯	◯	◯	◯	◯	Always

40. Evaluates of the whole learning process and looking at what I could have been done to improve performance helps development of competence. *

Mark only one oval.

	1	2	3	4	5	
Not	◯	◯	◯	◯	◯	Always

41. Learning online helps in the development of my competence *

Mark only one oval.

	1	2	3	4	5	
Not	◯	◯	◯	◯	◯	Always

42. Learning face to face helps in the development of my competence *

Mark only one oval.

	1	2	3	4	5	
Not	◯	◯	◯	◯	◯	Always

43. Learning using both face to face and online helps development of my competence *

Mark only one oval.

	1	2	3	4	5	
Not	◯	◯	◯	◯	◯	Always

44. Availability of clinical supply helps with development of competence *

Mark only one oval.

	1	2	3	4	5	
Not	◯	◯	◯	◯	◯	Always

45. Availability of clinical teachers help with development of my competence *

Mark only one oval.

	1	2	3	4	5	
Not	◯	◯	◯	◯	◯	Always

46. Availability of theoretical teachers helps with the development of my competence *

Mark only one oval.

	1	2	3	4	5	
Not	◯	◯	◯	◯	◯	Always

47. Availability of teaching nurses in the clinical areas helps with the development of my competence *

Mark only one oval.

	1	2	3	4	5	
Not	◯	◯	◯	◯	◯	Always

48. Availability of adequate nurses for patient care helps in the development of competence. *

Mark only one oval.

	1	2	3	4	5	
Not	◯	◯	◯	◯	◯	Always

49. Lecturers who are theoretically knowledgable help with the development of my competence *

Mark only one oval.

	1	2	3	4	5	
Not	◯	◯	◯	◯	◯	Always

50. Nurses who are theoretically knowledgable help with the development of my competence *

Mark only one oval.

	1	2	3	4	5	
Not	◯	◯	◯	◯	◯	Always

51. Lecturers who are clinically competent helps in the development of competence *

Mark only one oval.

	1	2	3	4	5	
Not	◯	◯	◯	◯	◯	Always

52. Nurses who are clinically competent helps in the development of competence *

Mark only one oval.

	1	2	3	4	5	
Not	◯	◯	◯	◯	◯	Always

....END..

THANK YOU FOR YOUR TIME

This content is neither created nor endorsed by Google.

Google Forms
